# Supplementary material for: Sequence and entropy-based control of complex coacervates
Source: Nat Commun. 2017 Nov 2;8:1273. doi: 10.1038/s41467-017-01249-1 (PMC5668414; doi:10.1038/s41467-017-01249-1)
Supplement: Supplementary file 1 — Supplementary Information [file 41467_2017_1249_MOESM1_ESM.pdf]

Supplementary Methods

Materials

Abbreviations for reagents are as follows: tert-butoxycarbonyl (Boc); 9-fluorenylmethoxylcarbonyl (Fmoc); t-butyl (tBu); trifluoroacetic acid (TFA); triisopropylsilane (TIPS); N,N-dimethylformamide (DMF); dichloromethane (DCM); N,N-diisopropylcarbodiimide (DIC); lysine (Lys or K); glutamate (Glu or E); glycine (Gly); ethyl (hydroxyimino)cyanoacetate (Oxyma).

Sequencing grade DMF, GC/MS grade DCM, TFA, ethyl ether anhydrous (BHT stabilized), methanol and acetonitrile (HPLC grade) were purchased from Fisher Scientific. Piperidine, α-Cyano-4-hydroxycinnamic acid, isopropanol (99%) was purchased from Sigma Aldrich. DIC (99%), TIPS (98%) was purchased from Acros Organics. Rink amide MBHA resin (loading level 0.32 mmol/g), Fmoc-*L*-Lys(Boc)-OH, Fmoc-*D*-Lys(Boc)-OH, Fmoc-*L*-Glu(tBu)-OH, Fmoc-*D*-Glu(tBu)-OH, Fmoc-Gly-OH, Oxyma were all purchased from Peptide Solutions, LLC. Contrad 70 was purchased from Decon Labs, Inc. All water was dispensed from a Milli-Q water purification system at a resistivity of 18.2 MΩ.cm.

Peptide Synthesis

Polypeptides were prepared using standard Fmoc-based solid-phase synthesis on a Liberty Blue automated microwave peptide synthesizer from CEM, Ltd.[1]. Deprotection and coupling were performed under microwave irradiation on a Rink amide MBHA resin with 0.2M Fmoc and Boc protected lysine (Fmoc-*L*-Lys(Boc)-OH, Fmoc-*D*-Lys(Boc)-OH), Fmoc and tBu protected glutamate (Fmoc-*L*-Glu(tBu)-OH, Fmoc-*D*-Glu(tBu)-OH), and Fmoc protected glycine (Fmoc-Gly-OH) in DMF. 20% Piperidine in DMF was used for Fmoc deprotection. DIC and Oxyma in a 0.5M and 1M concentration in DMF were used as activator and base, respectively.

Cleavage from the resin and side-chain deprotection was in performed using 10 mL of TFA/water/TIPS in the ratio of 95/2.5/2.5 for 3 hours at room temperature while bubbling with carbon dioxide The cleaved product and resin were separated by filtration. The crude peptide was then precipitated into 40 mL of cold (stored at -80°C) anhydrous ethyl ether. The mixture was then centrifuged for 5 min at 5,000 rpm (Sorvall Legend X1R Centrifuge, Thermo Fisher Scientific, lnc.). The supernatant was decanted and a second round of precipitation and centrifugation was performed. The crude product was then dried *in vacuo* in a desiccator overnight.

Characterization of the final product was performed via a Bruker UltrafleXtreme (Fremont, CA, USA) matrix-assisted laser desorption/ionization time of flight mass spectrometer (MALDI-TOF). Samples of the peptide were mixed with matrix solution (approximately 50 mg/mL α-cyano-4-hydroxycinnamic acid dissolved in 1:1 mixture of water and acetonitrile with 0.05% TFA) in 1:1 ratio to reach a final concentration of approximately 7.5 mM peptide.

Poly(glutamate) with degree of polymerization N = 50 was synthesized using amino acids of alternating chirality (*D* and *L*) to mitigate inter-peptide hydrogen bond formation.[2-4] Sequence-defined poly(lysine-co-glycine) peptides were synthesized with a degree of polymerization N = 50. Thus, all peptides include the charge-patterned blocks of 48 amino acids described by the block size τ, and are capped on each terminus by a single amino acid (K or G, Supplementary Table 1). For $\tau = 16$, the lysine portions of the peptide were synthesized using amino acids of alternating chirality (*D* and *L*) to mitigate inter-peptide hydrogen bond formation.[2-4] This use of alternating chirality was only implemented for the longest block size because of the tendency for complexing peptides to form β-sheets when a continuous run of more than 7-8 chiral amino acids is present.[3,4]

Supplementary Table 1. Molecular sequence for poly(lysine-co-glycine) peptides with degree of polymerization N = 50.

Block Size	Polypeptide Sequence
$\tau = 2$	$(KG)_{25}$
$\tau = 4$	$G(KKGG)_{12}K$
$\tau = 8$	$G(K_4G_4)_6K$
$\tau = 16$	$G(K_8G_8)_3K$

Coacervate Preparation

Polypeptide stock solutions were prepared gravimetrically using Milli-Q water at a concentration based on the total number of amino acids, as needed. For instance, a stock solution of poly(glutamic acid) of 10 mM amino acid would be used in parallel with a stock solution of the poly(lysine-co-glycine), also at 10 mM with respect to the total number of amino acids, or 5 mM with respect to the number of charged monomers present in solution. All solutions were adjusted to pH = 7.0 using concentrated solutions of HCl and NaOH, as needed. Monomer concentration was chosen as the experimental basis in order to easily enable direct stoichiometric comparison of the number of positively and negatively charged units present in solution, regardless of pH. A stock solution of NaCl was prepared gravimetrically at a concentration of 2 M and adjusted to pH = 7.0, as above.

Complexation was performed using stoichiometric quantities of positively and negatively charged polypeptides at a total charged residue concentration of 1 mM, 5 mM, and 50 mM at pH 7.0 (Figure 2b). Under these conditions, it is a reasonable approximation to describe all of the residues on both polypeptides as fully charged. Samples were prepared by first mixing a concentrated solution of NaCl with MilliQ water in a microcentrifuge tube (1.5 mL, Eppendorf), followed by the polyanion. The resulting mixture was then vortexed for 5 s before addition of the polycation (2 times of the volume as the polyanion to reach the charged balance between positively and negatively charged residue) to a final volume of 120 mL. The final mixture was vortexed for at least 15 s immediately after the addition of polycation to ensure fast mixing. The resulting phase separation causes the sample to take on a cloudy, or opalescent appearance, due to the formation of small droplets of the complex coacervate phase. Samples were then pipetted in triplicate into 384-well plates (FALCON, Corning, Inc.) with 32 μL per each well for subsequent analysis.

We also examined the effect of solvent dielectric by preparing samples at 1 mM total charged residue concentration in a 45/55 v/v% mixture of isopropanol and water (Supplementary Figure 1). Sample preparation and analysis was the same as described above, except that the mixture of isopropanol and water were combined first, followed by salt, and the various polypeptide solutions.

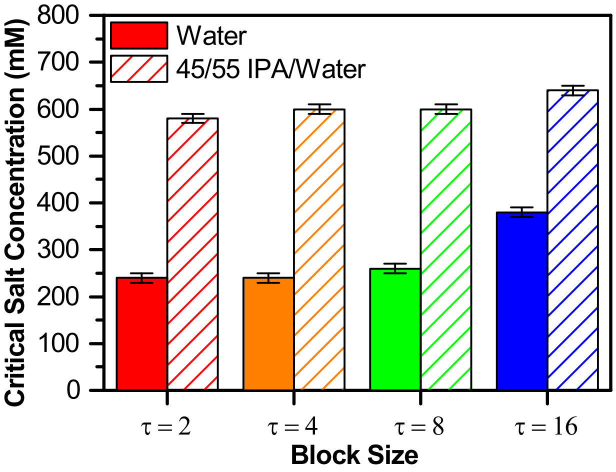

Supplementary Figure 1. The critical salt concentration (CSC) for sequence-defined coacervates at 1 mM charged monomer concentration in both water and a 45/55 v/v% mixture of isopropanol (IPA) and water.

Turbidimetry and Optical Microscopy

A plate reader equipped with a UV spectrophotometer (Synergy H1, BioTek, Inc.) was employed at a wavelength of 562 nm for the turbidity measurements. None of the polymers absorb light at this wavelength; thus, turbidity is due to light scattering from suspended coacervate droplets. The turbidity is defined by $-\ln(I/I_0)$, with I_0 = incident light intensity and I = intensity of light passed through the sample volume. Turbidity was used to estimate the critical salt concentration for a given sample. Direct examination of samples via optical microscope (EVOS XL Core, Fisher Scientific) was then used to confirm the critical salt concentration as the salt concentration above which no phase separation occurs. All samples were imaged within 1 h of preparation.

Isothermal Titration Calorimetry (ITC)

ITC experiments are performed at 25°C on a MicroCal Auto-iTC200 system (Malvern Instruments, Ltd.) All experiments were performed by injecting a 5 mM solution of the charge-patterned polycation (with respect to the number of lysines) into the sample cell containing 0.625 mM polyanion (with respect to the number of glutamates). Both solutions were prepared at a salt concentration of 25 mM NaCl and pH = 7.0 so as to minimize interference associated with heats of dilution. An initial injection of 0.5 µL was performed, followed by 3 injections of 2 µL each, 24 injections of 1 µL each, and then 4 injections of 2 µL each. This injection protocol was chosen to sample the various regions of the titration curve. An injection duration of 2 s followed by a 180 s equilibration time was used. Constant stirring speed is applied at a rate of 1000 rpm. All experiments were performed in triplicate.

The heat of dilution associated with injection of the charged-patterned polycation into the sample cell was measured in a separate reference experiment performed under identical conditions (pH = 7.0, 25mM NaCl), in the absence of polyanion in the cell. The measured heats of dilution were very small and were neglected in the subsequent data analysis.

Following each experimental run, a rigorous cleaning procedure was implemented. To clean the sample cell, the empty cell was briefly allowed to soak in a solution of 10% Contrad 70 detergent in water at 25°C. The pipette was cleaned by rinsing with water and methanol, followed by a drying step. Finally, the transfer tubing was cleaned with detergent, water, and methanol, followed by a drying step.

Analysis of ITC data was performed using the method reported previously by Priftis *et al*.[5]

ITC Data Analysis of Complex Coacervation

Initial Data Analysis by Microcal

Raw ITC data is in the form of the volume of material injected into the cell and the resultant power output in terms of µcal/injection. Given the volume of the cell (V_o), the volume of injectant added to the cell (ΔV), and the concentration of the solutions in the cell (C_{cell}) and in the syringe (C_{inj}) we can calculate all of the necessary parameters for data analysis.

The ITC cell itself is assumed to have a volume (V_o), which is completely full at the beginning of the experiment experiment (assume 200 µL for the ITC-200). A volume (ΔV) is added via injection from the syringe, and represents the excess volume of liquid that has overflowed the cell into the inactive communication tube where it does not participate further in the calorimetry experiment (

Supplementary Figure 2).

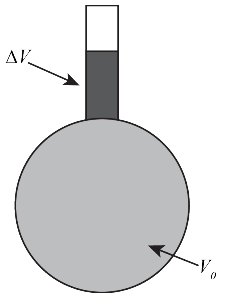

Supplementary Figure 2. Schematic of the ITC cell geometry. Figure adapted from the iTC-200 manual.

To begin with, it is necessary to calculate the concentration of the material injected by the syringe into the cell (X_t). For an injection (ΔV), the hypothetical concentration of injectant in the cell (X_t^o) is defined by the concentration of solution in the syringe and the characteristic volumes. This value is equal to the actual number of moles of injectant, modified by the number of moles of injectant lost into the overflow volume. Rather than using a straight mole balance, the Microcal manual describes the concentration in the overflow volume as the average between the starting concentration of the injectant in the cell (which is zero) and the hypothetical concentration.

$$X_t^o V_o = X_t V_o + \frac{1}{2} X_t^o \Delta V \tag{1}$$

Where

$$X_t^o = \frac{C_{inj} \Delta V}{V_o} \tag{2}$$

Rearranging Supplementary Eq. (1) gives an expression for X_t.

$$X_t = \frac{C_{inj} \Delta V}{V_o} \left(1 - \frac{\Delta V}{2V_o} \right) \tag{3}$$

Analysis of units for Supplementary Eq. (3) shows that X_t has the same units of concentration (mM) as does C_{inj}.

A similar analysis must be performed to determine the concentration of the species originally present in the cell (M_t) as it changes because of dilution from the injected volumes. In this case, M_t^o represents the concentration of material present in the cell at the beginning of the experiment and M_t is the concentration of material after the addition of injectant. As above, the amount of material lost into the excess volume is calculated based on an average concentration between M_t and M_t^o.

$$M_t^o V_o = M_t V_o + \frac{1}{2} \left(M_t + M_t^o \right) \Delta V \tag{4}$$

Rearranging Supplementary Eq. (4) gives a expression for M_t.

$$M_t = M_t^o \left(\frac{1 - \frac{\Delta V}{2V_o}}{1 + \frac{\Delta V}{2V_o}} \right) \tag{5}$$

The units of M_t are the same as M_t^o, and are concentration (mM).

The molar ratio of the injected species to that originally in the cell can thus be calculated from the values for X_t and M_t.

$$ratio = \frac{X_t}{M_t} \tag{6}$$

We then need to convert the experimentally measured heat values to enthalpies on a per mole basis. This is done by multiplying the measured enthalpy per injection by the volume of the injection and the concentration of the injectant and including unit conversions.

$$\frac{Enthalpy}{V_{inj} C_{inj}} \left(\frac{kJ}{mole} \right) \tag{7}$$

$$[=] \left(\frac{\mu cal}{injection} \right) \left(\frac{injection}{\mu L} \right) \left(\frac{L}{mmoles} \right) \left(\frac{10^6 \mu L}{L} \right) \left(\frac{10^3 mmoles}{moles} \right) \left(\frac{10^{-9} kJ}{0.23885 \mu cal} \right)$$

Two-Step Model for Complex Coacervation

Step One: Ion Pairing

Having obtained the data from the ITC instrument in a usable form, we now look to model coacervation as a two-step process. The first step, termed "Ion Pairing" is thought to involve the electrostatic complexation of soluble

polyelectrolyte chains. The resulting "ion pair" remains in solution and exhibits both enthalpy associated with the chain complexation and entropy associated with the release of bound counterions. This step is modeled using the "Single Set of Identical Sites" model from Microcal.

We begin by defining the complexation reaction:

$$X + M \underset{k_{off}}{\overset{k_{on}}{\rightleftharpoons}} XM \tag{8}$$

Where the association binding constant (K_a) is defined as

$$K_a = \frac{k_{on}}{k_{off}} = \frac{[MX]}{[M][X]} \tag{9}$$

where [M], [X], and [MX] are the concentrations of the unbound species in the cell and syringe, and the concentration of the ion paired complex, respectively.

However, only the total concentrations X_t and M_t are known. We must therefore eliminate [M], [X], and [MX] from our expressions. A total mole balance for both M and X, taking into account the stoichiometry of X binding to M (n_{IP}) gives:

$$X_t = [X] + n_{IP}[MX] \tag{10}$$

$$M_t = [M] + [MX] \tag{11}$$

We can also define the fraction of sites on M that are bound in an ion pair (Θ_{IP}).

$$\Theta_{IP} = \frac{[MX]}{M_t} \tag{12}$$

Combining Supplementary Eqs. (11) and (12) we can write

$$[M] = M_t(1 - \Theta_{IP}) \tag{13}$$

Similarly, combining Supplementary Eqs. (10) and (12) we obtain

$$[X] = X_t - n_{IP}\Theta_{IP}M_t \tag{14}$$

Rearranging Supplementary Eq. (12) gives us an expression for the remaining unknown parameter, [MX]

$$[MX] = \Theta_{IP}M_t \tag{15}$$

Finally, substituting the expressions from Supplementary Eqs. (13) and (15) into the expression for the association binding constant Supplementary Eq. (9) gives

$$K_a = \frac{\Theta_{IP}}{(1 - \Theta_{IP})[X]} \tag{16}$$

Further including Supplementary Eq. (14) allows for the creation of an equation where the only unknown is Θ_{IP}.

$$K_a = \frac{\Theta_{IP}}{(1 - \Theta_{IP})(X_t - n_{IP}\Theta_{IP}M_t)} \tag{17}$$

Expanding the quadratic and gathering terms we obtain

$$\Theta_{IP}^2 - \Theta_{IP}\left(1 + \frac{X_t}{n_{IP}M_t} + \frac{1}{K_a n_{IP} M_t}\right) + \frac{X_t}{n_{IP}M_t} = 0 \tag{18}$$

Solving this expression for Θ_{IP} gives

$$\Theta_{IP}=\frac{1}{2}\left[1+\frac{X_t}{n_{IP}M_t}+\frac{1}{K_a n_{IP}M_t}-\sqrt{\left(1+\frac{X_t}{n_{IP}M_t}+\frac{1}{K_a n_{IP}M_t}\right)^2-\frac{4X_t}{n_{IP}M_t}}\right] \tag{19}$$

The choice of this solution for Θ_{IP} is straightforward because it provides the only physically meaningful answer (at low X_t and M_t the value of Θ_{IP} is small and asymptotically approaches 1 at large X_t and M_t.

The total heat content Q_{IP} of the solution in the cell (determined relative to zero for the unbound species) at a fractional saturation of Θ_{IP} and using a characteristic change in enthalpy for ion pairing (ΔH_{IP}) is:

$$Q_{IP}=\frac{n_{IP}\Theta_{IP}M_t\Delta H_{IP}V_o}{C_{inj}\Delta V} \tag{20}$$

which has units of kJ mol^{-1}.

$$Q_{IP}[=]\left(\frac{mmoles}{L}\right)\left(\frac{kJ}{mole}\right)\left(\frac{\mu L}{}\right)\left(\frac{L}{mmoles}\right)\left(\frac{}{\mu L}\right) \tag{21}$$

Substituting Supplementary Eq. (19) into Supplementary Eq. (20) gives the full expression for the molar heat of ion pairing.

$$Q_{IP}=\frac{n_{IP}M_t\Delta H_{IP}V_o}{2C_{inj}\Delta V}\left[1+\frac{X_t}{n_{IP}M_t}+\frac{1}{K_a n_{IP}M_t}-\sqrt{\left(1+\frac{X_t}{n_{IP}M_t}+\frac{1}{K_a n_{IP}M_t}\right)^2-\frac{4X_t}{n_{IP}M_t}}\right] \tag{22}$$

However, in modeling our experimental data we are interested in the change in heat from one injection to the next (ΔQ_{IP}). This calculation requires consideration of the values for Q_{IP} and ΔV after the ith injection. We are also only considering the heat effects within the cell volume V_o, necessitating the inclusion of a correction term as in Supplementary Eqs. (1) and (4).

$$\Delta Q_{IP}(i)=Q_{IP}(i)-Q_{IP}(i-1)+\frac{\Delta V}{V_o}\left(\frac{Q_{IP}(i)+Q_{IP}(i-1)}{2}\right) \tag{23}$$

Step Two: Complex Coacervation

Following the formation of soluble "ion pairs," we now describe a second step where a molecular rearrangement occurs, allowing the soluble ion pairs to aggregate and undergo phase separation to form the separate liquid coacervate phase. For this step we assume that the additional heat contribution to the system (Q_{coac}) is proportional to the amount of polymer present in the coacervate domains at a given composition. We define

$$Q_{coac}=\frac{n_{coac}\Theta_{coac}M_t\Delta H_{coac}V_o}{C_{inj}\Delta V} \tag{24}$$

Where Θ_{coac} is the fraction of sites involved in coacervation and is arbitrarily defined by a Gaussian with respect to the molar fraction f of the polymer being titrated into the system over the total polymer content. This choice was made because the Gaussian shape is similar to the shape of a turbidity curve as a function of polyelectrolyte stoichiometry.

$$f=\frac{X_t}{X_t+M_t} \tag{25}$$

$$\Theta_{coac} = \exp\left(-\frac{\left(f - f_{coac}\right)^2}{\alpha_{coac}^2} \right) \tag{26}$$

The parameter f_{coac} describes the composition of the mixture at the point of maximum coacervate formation in terms of the stoichiometry of binding n_{coac}.

$$f_{coac} = \frac{n_{coac}}{1 + n_{coac}} \tag{27}$$

α is related to the magnitude of the full-width at half-maximum (FWHM) for the Gaussian curve.

$$FWHM = 2\alpha\sqrt{\ln(2)} \approx 1.7\alpha \tag{28}$$

The change in heat for the coacervation step is calculated as a difference between titration steps, as above.

$$\Delta Q_{coac}(i) = Q_{coac}(i) - Q_{coac}(i-1) + \frac{\Delta V}{V_o}\left(\frac{Q_{coac}(i) + Q_{coac}(i-1)}{2} \right) \tag{30}$$

This two-step model is intended to be used to fit experimentally obtained data from isothermal titration calorimetry (ITC). It consists of six fitting parameters: n_{IP}, n_{coac}, ΔH_{IP}, ΔH_{coac}, K_a, and α. Here are some suggestions for starting values for these parameters. n_{IP} corresponds to the ratio where the ion pairing transition (inflection point) occurs. This is typically near the middle of the range of ratios run for the experiment (assuming your data fills the entire range). n_{coac} corresponds to the ratio where the curve crosses the horizontal axis. All ratios below n_{coac} have a positive enthalpy change and all ratios above n_{coac} have a negative one (or visa versa). In physical terms, while adding a small amount of the injectant, you form more and more coacervate until you reach the ratio n_{coac}. After that, each injection causes the coacervate to gradually dissolve. This is also why n_{coac} is the ratio at which we observe the largest amount of coacervation formation. Typically this value is slightly higher than n_{IP}. ΔH_{IP} corresponds to the horizontal asymptote for the experimental data for the first couple of points during the titration experiment. ΔH_{coac} is a normalization value. While we would prefer to directly utilize the change in enthalpy per mole of polymer that is actually present in the coacervate phase, we have no way of determining this value. A starting guess is typically 10-20% of the value of ΔH_{IP}. K_a corresponds to the steepness of the slope of the overall curve (i.e. at infinitely high K you would get a step function). Suggested starting values are in the range of 10^2 mM^{-1}. α corresponds to the width of the range over which coacervation occurs. It is directly proportional to the distance between the minimum and the maximum of ΔQ_{coac}. Typical starting value is approximately 0.02.

Analysis of Thermodynamic Parameters

The two-step model directly provides values for enthalpy of ion pairing and coacervation (ΔH_{IP}, ΔH_{coac}) as well as the association binding constant for the ion pairing step (K_a). From these parameters we can determine values for the Gibbs free energy (ΔG_{IP}) and the entropy (ΔS_{IP}) associated with ion pairing. By definition, the association binding constant is directly related to the Gibbs free energy.

$$\Delta G_{IP} = -RT\ln\left(K_a\right) \tag{31}$$

We can now calculate the value for the entropy (ΔS_{IP}) using the definition for the Gibbs free energy.

$$\Delta G_{IP} = \Delta H_{IP} - T\Delta S_{IP} \tag{32}$$

$$\Delta S_{IP} = \frac{\Delta H_{IP} - \Delta G_{IP}}{T} \tag{33}$$

Data Analysis

Reported data are the average of three runs. An average of the last five points of each run was used to correct the baseline of each dataset. This approach assumes that the system has reached equilibrium by the end of the run, and that the observed change in enthalpy is zero.

ITC data analysis was performed in Microsoft Excel. Curve fitting to the model was performed via least-squared error minimization with six adjustable parameters (ΔH_{ip}, K_a, n_{ip}, ΔH_{coac}, α, n_{coac}) using the Solver add-on. Supplementary Figure 3 shows the results of fitting the two-step model to our data. The single binding-site model used for the ion pairing step results in a smooth continuously decaying function (red line). The coacervation step shows the characteristic features of the derivative of a Gaussian peak, reflective of the formation and subsequent dissolution of the coacervate phase as a function of the mole ratio of the system. Supplementary Figure 4 shows a comparison of the data and the resulting curve fits as a function of block size.

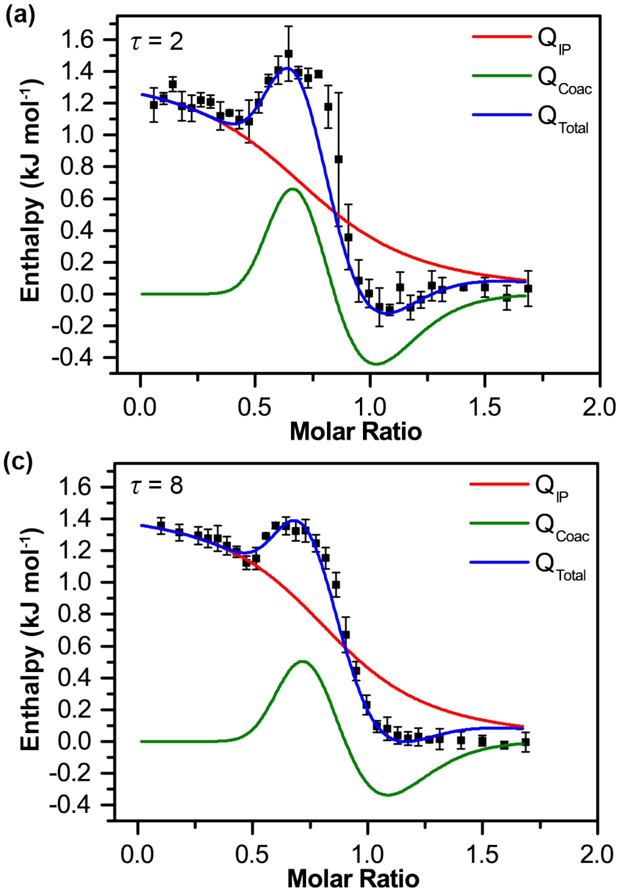

Supplementary Figure 3. Experimental data for the molar enthalpy of complexation of sequence-patterned polypeptides in 25 mM NaCl and pH = 7.0 and the resulting fitting curves (blue line) for **(a)** $\tau = 2$, **(b)** $\tau = 4$, **(c)** $\tau = 8$, and **(d)** $\tau = 16$. The individual contributions from the ion pairing (red line) and coacervation (green line) parts of the model are shown in each graph. Values represent the average of three runs; error bars are the standard deviation.

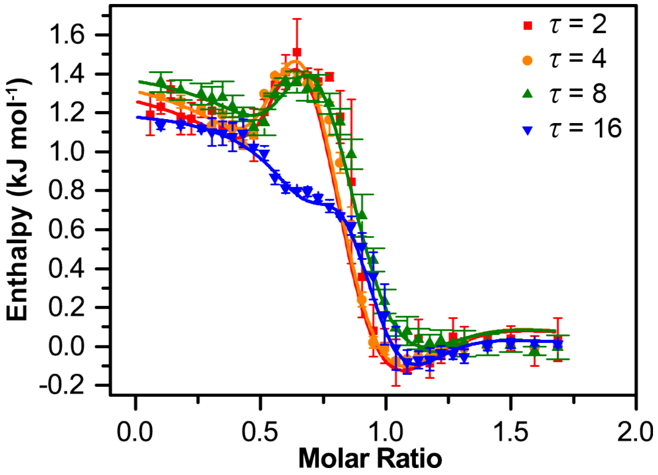

Supplementary Figure 4. Results of curve fitting superimposed onto the raw ITC data for sequence-patterned polypeptides in 25 mM NaCl and pH = 7.0. Values represent the average of three runs; error bars are the standard deviation.

Supplementary Table 2. Fitting parameters for ITC analysis for sequence-patterned polypeptides in 25 mM NaCl and pH = 7.0. Values represent the average of three runs. Calculated error is the result of curve fitting based on the minimum and maximum variation from the average defined by the standard deviation.

Block Size	ΔH_{ip} (kJ mol^{-1})	K_a (L mmol^{-1})	n_{ip}	ΔH_{coac} (kJ mol^{-1})	α	n_{coac}
$\tau = 2$	1.38±0.074	18.5±0.11	0.80±0.014	0.196±0.0013	0.077±0.0017	0.85±0.036
$\tau = 4$	1.45±0.024	18.6±0.63	0.82±0.010	0.196±0.0010	0.077±0.0002	0.85±0.036
$\tau = 8$	1.46±0.004	25.0±0.62	0.89±0.008	0.143±0.0019	0.074±0.0009	0.90±0.011
$\tau = 16$	1.22±0.010	58.8±0.12	0.74±0.006	0.058±0.0001	0.051±0.0005	0.96±0.025

Supplementary Table 3. Compiled thermodynamic data from ITC analysis for sequence-patterned polypeptides in 25 mM NaCl and pH = 7.0. Values represent the average of three runs. ΔH values are the sum of ΔH_{ip} and ΔH_{coac}.

Block Size	ΔH (kJ mol^{-1})	$-T\Delta S$ (kJ mol^{-1})	ΔG (kJ mol^{-1})
$\tau = 2$	1.58±0.075	–25.7±0.09	–24.3±0.01
$\tau = 4$	1.64±0.025	–25.8±0.06	–24.3±0.08
$\tau = 8$	1.60±0.006	–26.4±0.06	–25.1±0.06
$\tau = 16$	1.28±0.010	–28.4±0.01	–27.2±0.01

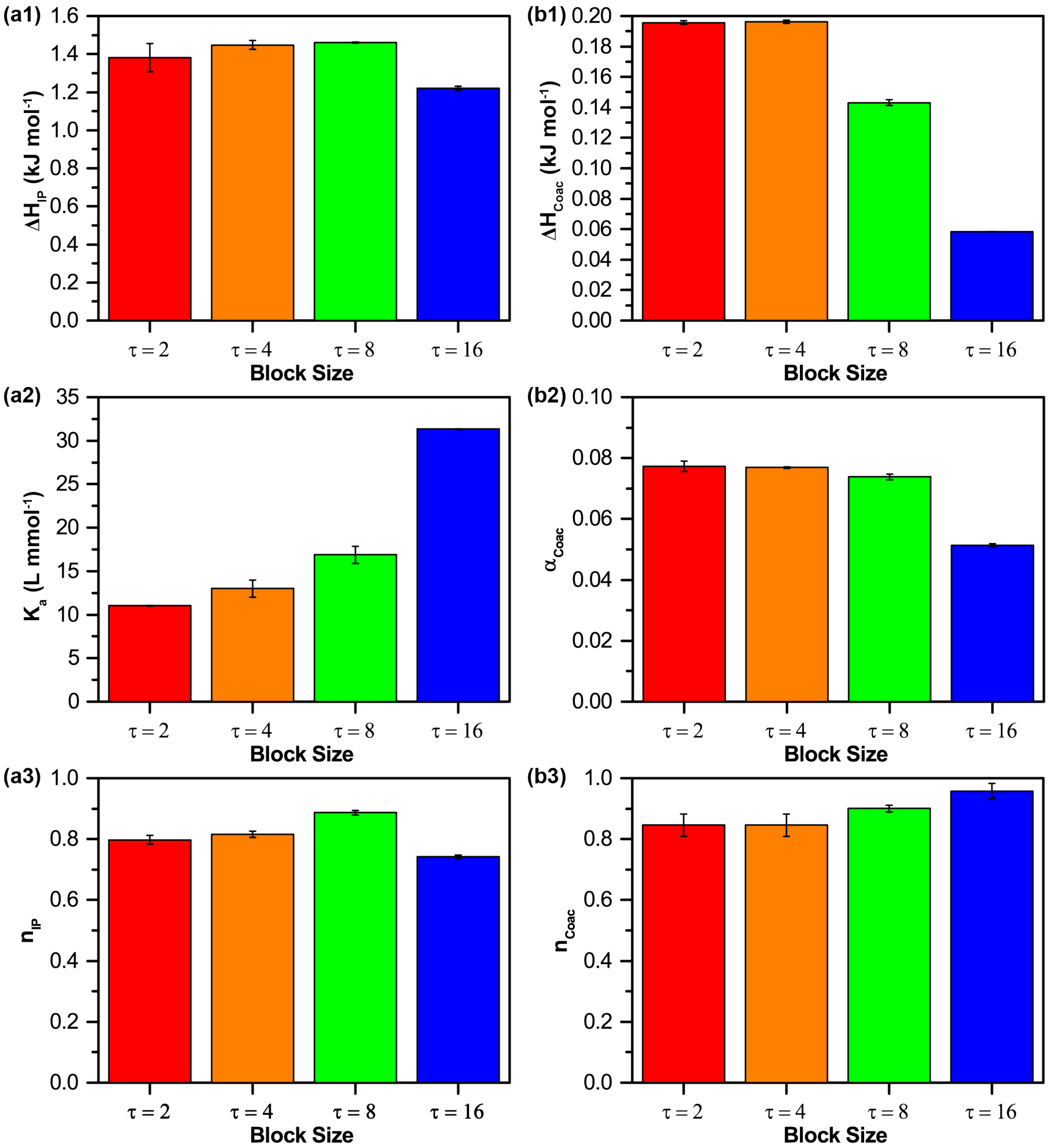

Supplementary Figure 5. Fitting parameters from ITC analysis for the **(a)** ion pairing and **(b)** coacervation steps for sequence-patterned polypeptides in 25 mM NaCl and pH = 7.0. Values represent the average of three runs. Error bars are the result of curve fitting based on the minimum and maximum variation from the average defined by the standard deviation.

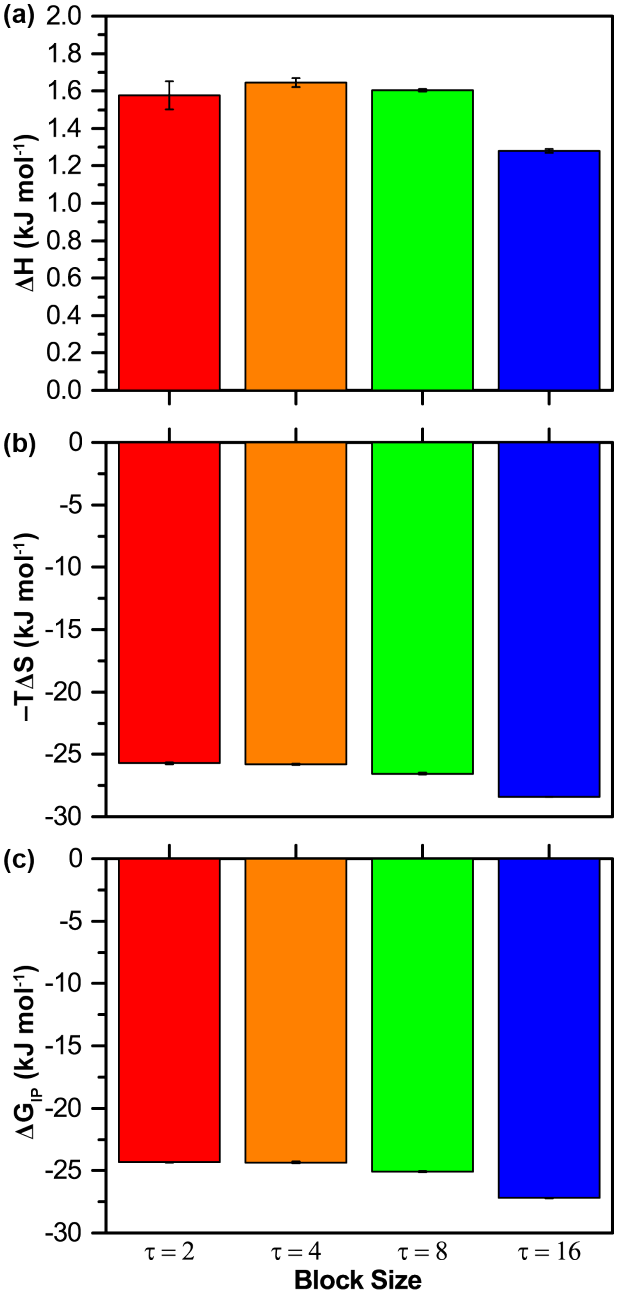

Supplementary Figure 6. Compiled thermodynamic data from ITC data analysis for sequence-patterned polypeptides in 25 mM NaCl and pH = 7.0. Values represent the average of three runs. ΔH values are the sum of ΔH_{ip} and ΔH_{coac}.

Simulation Determination of Coacervate Phase Behavior

Our approach for simulating complex coacervation is related to methods developed in Lytle, et al.[6] that use Monte Carlo (MC) simulations to tabulate the excess free energy, $f_{EXC}(\phi_P, \phi_S)$ as a function of polymer volume fraction ϕ_P and salt volume fraction ϕ_S. This molecularly-informed function f_{EXC} can be incorporated into a Flory-Huggins like free energy expression, which can subsequently be used to calculate the phase behavior of the coacervation for a series of sequence-defined polycations and homopolyanions.

Restricted Primitive Model of Coacervation

MC simulations were performed in the NVT ensemble using the restricted primitive model (RPM),[7] which treats all charged species as beads that interact via hard core potentials and Coulombic interactions. Systems composed of n_{P+} polycations, n_{P-} polyanions, n_+ cations, and n_- anions at positions $\boldsymbol{r}_i$. Water is modeled as an implicit solvent with a relative dielectric constant $\epsilon_r = 78.5$. We fix the degree of polymerization, N, to be 48 beads for all chains. Polycations are patterned with 24 neutral beads and 24 charged beads. n_{P+} is twice the value of n_{P-} to ensure the polymers are charge neutral without additional salt. Charge beads, including all salt ions and polymer charges, have a hard-core diameter $\sigma_{P+} = \sigma_{P-} = \sigma_+ = \sigma_- = 4.25$ A. Neutral beads have smaller diameters $\sigma_0 = 0.25\sigma_+$, motivated by the absence of a hydration shell that is implicitly included in the hard-core radius of the charged species in the RPM. Practically, the neutral beads' size does have a measurable effect on the magnitude of coacervate phase behavior; we have parameterized this value to match experimental and computational phase behavior.

MC Simulation of Coacervates

The MC simulation is updated based on the overall interaction energy U given by:

$$U = \frac{1}{2}\sum_{i,j\neq i}^{N_{tot}}\left[U_{HS}(r_{ij}) + U_{ES}(r_{ij})\right] + \sum_{i}^{n}\sum_{j}^{N-1} U_B(r_{j,j+1}) + \sum_{i}^{n}\sum_{j}^{N-2} U_\theta(\boldsymbol{r}_j, \boldsymbol{r}_{j+1}, \boldsymbol{r}_{j+2}) \tag{34}$$

where $N_{tot} = N(n_{P+} + n_{P-}) + n_+ + n_-$, the total number of beads, and $n = n_{P+} + n_{P-}$ is the total number of polymer chains. $r_{ij} = |\boldsymbol{r}_i - \boldsymbol{r}_j|$ is the distance between beads i and j. The hard sphere interaction contribution, U_{HS}, is given by:

$$U_{HS}(r_{ij}) = \begin{cases} \infty \; if \; r_{ij} < (\sigma_i + \sigma_j)/2 \\ 0 \; if \; r_{ij} \geq (\sigma_i + \sigma_j)/2 \end{cases} \tag{35}$$

The polymer bonding potential contribution U_B is:

$$U_B(r_{ij}) = \begin{cases} 0 \;\; if \; \sigma_+ \leq r_{ij} \leq 1.1\sigma_+ \\ \infty \qquad otherwise \end{cases} \tag{36}$$

Here, the polymer bonds are all the same size regardless of the actual diameter of the monomer σ_i, and is set by the size of the polycation/polyanion bead. An angle potential contribution U_θ provides some stiffness for the chain:

$$U_\theta(\boldsymbol{r}_j, \boldsymbol{r}_{j+1}, \boldsymbol{r}_{j+2}) = \frac{1}{2}\kappa_\theta \theta_{j,j+1,j+2}^2 \tag{37}$$

where $\kappa_\theta = 3.3k_BT$ is the strength of the angle potential, k_B is the Boltzmann constant, T is the temperature, and $\theta_{j,j+1,j+2}$ is the angle between adjacent bond vectors. Finally, all charged beads interact through a Coulomb potential U_{ES}:

$$U_{ES}(r_{ij}) = \frac{q_i q_j}{4\pi\epsilon_0\epsilon_r r_{ij}} \tag{38}$$

where q_i is the charge on bead i, and ϵ_0 is the vacuum permitivitty. Ewald summation is used to calculate electrostatic interactions in the simulation.[8]

Widom Insertion to Calculate Free Energy Landscape

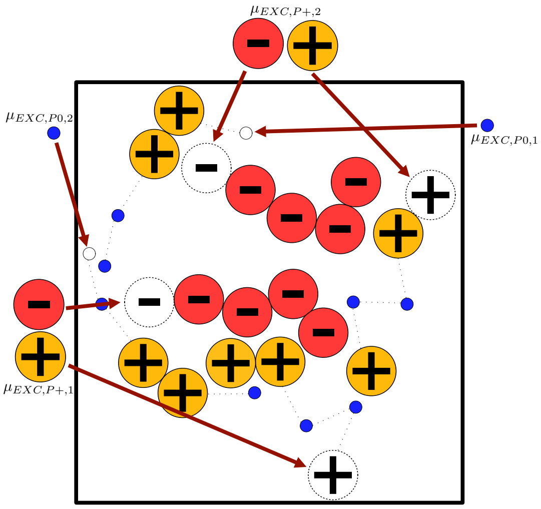

Supplemental Figure 7: Example calculation for the polymer excess chemical potential with a $\tau = 4$ repeating pattern. Charged polycation monomers are orange, neutral polycation monomers are blue, and polyanion monomers are red. Bonds are denoted by dotted lines. Sequence-defined polycation chains are generated such that each bead in the repeating pattern is represented by a chain end. In this case, polycation chain ends correspond to the first charged bead, the second charged bead, the first neutral bead, and the second netural bead. Widom insertion is then performed on the different chain ends, and, if the inserted polycation monomer is charged then a polyanion monomer is also inserted. For this particular pattern, the four excess chemical potentials are $\mu_{EXC,P+,1}$, $\mu_{EXC,P+,2}$, $\mu_{EXC,P0,1}$, $\mu_{EXC,P0,2}$. These correspond to the insertion of the first charged polycation monomer with a polyanion monomer, the second charged polycation monomer with a polyanion monomer, the first neutral polycation monomer, and the second neutral polycation monomer, respectively. These excess chemical potentials are added together and divided by the total number of monomers inserted for all excess chemical potentials. This scheme can be generalized to any pattern size.

The excess chemical potentials, $\mu_{EXC,i}$ for all species $i = P+, P-, +, -, 0$ were calculated using Widom insertion.[8] Modification of the Widom insertion technique was necessary to calculate the polyelectrolytes' excess chemical potential, illustrated schematically in Supplemental Figure 7. The polycation's pattern is shifted along the various chains in the system so each monomer in the charge pattern has representative chain ends. Widom insertion is performed on each of the diferent types of chain ends, and, if the inserted polycation monomer is charged, a corresponding polyanion monomer is inserted. The chemical potentials thus calculated are added together and divided by the total number of monomers inserted for all of the chain ends. This gives each monomers' excess chemical potential. The salt excess chemical potential $(\mu_{EXC,+} + \mu_{EXC,-})/2$ was calculated by inserting a pair of oppositely-charged salt ions and halving this value, yielding the excess chemical potential for each salt ion. Thermodynamic integration of $\mu_{EXC,i}$ according to:

$$f_{EXC}(\phi_S,\phi_P) = \int_{\phi_S^0,\phi_P^0}^{\phi_S,\phi_P^0} \mu_{EXC,S}(\phi_S',\phi_P^0)d\phi_S{}' + \int_{\phi_S,\phi_P^0}^{\phi_S,\phi_P} \mu_{EXC,P}(\phi_S,\phi_P{}')d\phi_P{}' \tag{39}$$

This yields the excess free energy density, $f_{EXC}(\phi_S,\phi_P)$, as a function of ϕ_S and ϕ_P. This excess free energy density is used in a Flory-Huggins-like theory to determine the phase behavior of the system.

Phase Separation Theory

A Flory-Huggins inspired theory was developed to determine the coacervation phase diagrams. The system has an average polymer volume fraction, $\langle\phi_P\rangle$, and an average salt volume fraction, $\langle\phi_S\rangle$. At certain salt and polymer volume fractions, phase separation occurs creating a coacervate phase, α, with polymer volume fraction, $\phi_{P,\alpha}$, and salt volume fraction, $\phi_{S,\alpha}$. Coexisting with the coacervate phase is a supernatant phase, β, with polymer volume fraction $\phi_{P,\beta}$ and salt volume fraction $\phi_{S,\beta}$. Both phases are incompressible with a volume fraction of water given by $\phi_{W,i} = 1 - \phi_{P,i} - \phi_{S,i}$ where i denotes the phase. The free energy of the system is:

$$F = F_0 + F_{EXC} \tag{40}$$

Here, F_0 is the translational entropy contribution to the overall free energy, and F_{EXC} is the excess free energy contribution.

The entropy of mixing is given by:

$$\begin{aligned} F_0 = \frac{\Omega}{\langle\phi_P\rangle}\Bigg\{&\left[1-\frac{pN\langle\phi_P\rangle}{\Omega\phi_{P,\alpha}}\right]\left[\phi_{S,\beta}\ln\phi_{S,\beta} + \frac{\phi_{P,\beta}}{N}\ln\phi_{P,\beta} + \left(1-\phi_{P,\beta}-\phi_{S,\beta}\right)\ln\left(1-\phi_{P,\beta}-\phi_{S,\beta}\right)\right] \\ &+ \frac{pN\langle\phi_P\rangle}{\Omega\phi_{P,\alpha}}\Bigg[\left(1-\phi_{P,\alpha}-\phi_{S,\alpha}\right)\ln\left(1-\phi_{P,\alpha}-\phi_{S,\alpha}\right) + \phi_{S,\alpha}\ln\phi_{S,\alpha} \\ &+ \frac{\phi_{P,\alpha}}{N}\ln\phi_{P,\alpha}\Bigg]\Bigg\} \end{aligned} \tag{41}$$

Here, Ω is the total number of monomers in the system, and p is the total number of chains in the coacervate phase. The first term describes the entropy of mixing in the supernatant phase, and the second term describes the entropy of mixing in the coacervate phase. $\frac{pN\langle\phi_P\rangle}{\Omega\phi_{P,\alpha}}$ gives the ratio of the coacervate phase volume to the total system volume, and $\Omega/\langle\phi_P\rangle$ gives the total number of monomer-equivalent volumes in the system.

The excess free energy contribution is given by:

$$F_{EXC} = \frac{\Omega}{\langle\phi_P\rangle}\left\{\left[1-\frac{pN\langle\phi_P\rangle}{\Omega\phi_{P,\alpha}}\right]f_{EXC}\left(\phi_{P,\beta},\phi_{S,\beta}\right) + \frac{pN\langle\phi_P\rangle}{\Omega\phi_{P,\alpha}}f_{EXC}\left(\phi_{P,\alpha},\phi_{S,\alpha}\right)\right\} \tag{42}$$

Here, f_{EXC} is the excess free energy density obtained in Supplemental Equation 39. This allows any monomer-level features, such as the patterning to be included in the mixing enthalpy.

F is minimized with respect to $\phi_{P,\alpha}$, $\phi_{P,\beta}$, and $\phi_{S,\alpha}$. p can be calculated using $\langle\phi_P\rangle = \frac{pN\langle\phi_P\rangle}{\Omega\phi_{P,\alpha}}\phi_{P,\alpha} + \left(1 - \frac{pN\langle\phi_P\rangle}{\Omega\phi_{P,\alpha}}\right)\phi_{P,\beta}$ and the corresponding calculation for $\langle\phi_S\rangle$ can be used to calculate $\phi_{S,\beta}$. The minimized value of F is compared to the free energy of a homogeneous state, F_{HOM}, given by:

$$F_{HOM} = \frac{\Omega}{\langle\phi_P\rangle}\Big[\langle\phi_S\rangle\ln\langle\phi_S\rangle + (1 - \langle\phi_P\rangle - \langle\phi_S\rangle)\ln(1 - \langle\phi_P\rangle - \langle\phi_S\rangle) + \frac{\langle\phi_P\rangle}{N}\ln\langle\phi_P\rangle + f_{EXC}(\langle\phi_P\rangle, \langle\phi_S\rangle)\Big] \tag{43}$$

If the minimized value of F is less than F_{HOM}, the system undergoes phase separation to the concentration values which minimized F.

Supplementary Note 1

Along-the-chain Correlation Functions

We characterize the structure and sequence behaviors of charge sequences using a pair of correlation functions C_1 and C_2 that characterize spatial and sequence-based structure respectively. Both consider an initial pair of oppositely-charged monomers, i and j, that are separated by a distance less than or equal to a cutoff r_C. We then characterize properties of two beads a constant number of monomers Δs along the chain contour, $i + \Delta s$ and $j + \Delta s$.

$C_1(\Delta s)$ provides a structural measure of looping among neighboring polyelectrolytes. We calculate the probability that $i + \Delta s$ and $j + \Delta s$ are also within the cutoff r_C, *if* both monomers are charged. Formally, this is given by the equation:

$$C_1(\Delta s) = \frac{\langle\sum_i\sum_j \delta(z_i - 1)\delta(z_j + 1)\delta(z_{i+\Delta s} - 1)\delta(z_{j+\Delta s} + 1)\Theta(r_C - r_{ij})\Theta(r_C - r_{i+\Delta s,j+\Delta s})\rangle}{\langle\sum_i\sum_j \delta(z_i - 1)\delta(z_j + 1)\delta(z_{i+\Delta s} - 1)\delta(z_{j+\Delta s} + 1)\Theta(r_C - r_{ij})\rangle} \tag{44}$$

Here the function $\Theta(x)$ is the Heaviside function that is $\Theta(x) = 1$ for $x \geq 0$ and $\Theta(x) = 0$ for $x < 0$. The average denoted by the angle brackets $\langle\cdots\rangle$ represents ensemble averages taken over the course of a simulation, and $\delta(x)$ is the Dirac delta function. This is a measure of conformational correlations by determining the subset of polyelectrolyte charges that loop over a number of monomers Δs.

This measure of $C_1(\Delta s)$ between charged particles has some values that are necessarily 0 due to the periodicity of the pattern; these are removed from representations of this function for clarity.

$C_2(\Delta s)$ is a related measure of the sequence correlations:

$$C_2(\Delta s) = \frac{\langle\sum_i\sum_j \delta(z_i - 1)\delta(z_j + 1)\delta(z_{i+\Delta s} - 1)\delta(z_{j+\Delta s} + 1)\Theta(r_C - r_{ij})\Theta(r_C - r_{i+\Delta s,j+\Delta s})\rangle}{\langle\sum_i\sum_j \delta(z_i - 1)\delta(z_j + 1)\Theta(r_C - r_{ij})\Theta(r_C - r_{i+\Delta s,j+\Delta s})\rangle} \tag{45}$$

The difference in this correlation function is that we are now considering the subset of loops that consist of charged monomers. For this work, we set $r_C = 1.5\sigma_+$.

Supplementary Note 2

One-dimensional Adsorption Model

We can use simulation data of a single, dilute polyelectrolyte chain in a salt solution to calculate the entropic driving force for counterion release. To do this, we map simulation data to a one-dimensional adsorption model where each monomer of the polyelectrolyte chain is a 'site' that can contain a condensed counterion. These adsorbed counterions are in equilibrium with the external solution that is a constant chemical potential μ reservoir of salt ions. Each adsorbed ion 'feels' an effective binding energy ϵ_i that is due to the electrostatic characteristics of the chain and the surrounding condensed charges, and is a function of the chain index i. The grand canonical partition function for adsorption on to a chain of length N is thus:

$$\Xi = \prod_{i}^{N} \left(1 + e^{-\beta(\epsilon_i - \mu)}\right) \tag{46}$$

Standard statistical mechanics leads to expressions for both the average number of adsorbed counterions $\langle n_i \rangle$ at a given index i and the overall entropy of the adsorbed counterions S_{ads}:

$$\sum_i \langle n_i \rangle = k_B T \left(\frac{\partial \ln \Xi}{\partial \mu}\right)_T = \sum_i \frac{e^{-\beta(\epsilon_i - \mu)}}{1 + e^{-\beta(\epsilon_i - \mu)}} \tag{47}$$

$$S_{ads} = k_B \left(\frac{\partial T \ln \Xi}{\partial T}\right)_{\tilde{\mu}} = k_B \left[\sum_i \ln\left(1 + e^{-\beta(\epsilon_i - \mu)}\right) + \frac{\epsilon_i}{k_B T}\left(\frac{e^{-\beta(\epsilon_i - \mu)}}{1 + e^{-\beta(\epsilon_i - \mu)}}\right)\right] \tag{48}$$

This calculation requires determining the parameters of this model; namely, the values of the energy ϵ_i along the chain and the chemical potential of the reservoir. We use simulation to determine the former, and keep the latter as a parameter that is constant for all systems at a given salt concentration.

Determination of Counterion Condensation

We first determine the number and distribution of condensed counterions. We use a methodology described in Liu and Muthukumar[9] to characterize the extent of counterion condensation along a dilute polyelectrolyte chain. In this methodology, a cutoff distance r_{CC} is chosen to represent the near-chain region; salt ions that are within r_{CC} from a polyelectrolyte bead are considered condensed. We choose $r_{CC} = 1.5\sigma_{P+}$. This is somewhat arbitrary, however we find that our results are not strongly affected by the specific choice of r_{CC}. We schematically demonstrate this method in Figure 5b. Condensed counterions are assigned to an index i, which is the nearest polyelectrolyte chain monomer. Averaged over a simulation run, we obtain a value of $\langle n_i \rangle$.

The size difference between charged and neutral beads causes the value of $\langle n_i \rangle$ to be significantly different for a fixed r_{CC}. $\langle n_i \rangle$ for a charged bead will typically be smaller than for a neutral bead because there is less unoccupied volume. This does not, however, represent a physically meaningful difference, but rather due to the arbitrary definition of r_{CC}. Rather than vary r_{CC}, we choose to normalize $\langle n_i \rangle$ to remove this disparity by calculating a value $\langle n_i^0 \rangle$ that is determined from simulations where the polyelectrolyte charge is taken to $z_{P+} = 0$. This establishes the number of counterions that would fit the definition of a condesned charge for a neutral chain, which also varies with the different-sized beads. Indeed, this variation introduces the same effect as for a charged chain, so a ratio $\langle n_i \rangle / \langle n_i^0 \rangle$ removes effects due to the difference in unoccupied volumes between the two bead types.

Effective Binding Energy

We can convert the number of counterion beads into an effective ϵ_i. We do so using the previously determined relationship:

$$\langle n_i \rangle = \frac{e^{-\beta(\epsilon_i - \mu)}}{1 + e^{-\beta(\epsilon_i - \mu)}} \tag{49}$$

The effective energy ϵ_i is defined as the effect of the charged polyelectrolyte chain and the condensed counterions. We can thus set $\epsilon_i \to 0$ for the uncharged polyelectrolyte chains, leading to the relationship:

$$\langle n_i^0 \rangle = \frac{e^{\beta\mu}}{1 + e^{\beta\mu}} \tag{50}$$

We can thus define the ratio:

$$\frac{\langle n_i \rangle}{\langle n_i^0 \rangle} = \frac{\left(1 + e^{\beta\mu}\right) e^{-\beta(\epsilon_i - \mu)}}{\left(1 + e^{-\beta(\epsilon_i - \mu)}\right) e^{\beta\mu}} \tag{51}$$

The quantities $e^{\beta\mu} \ll 1$ and $e^{-\beta(\epsilon_i - \mu)} \ll 1$ for the cases we consider in this paper (an *a posteriori* observation). We can thus simplify the relationship to the following expression for the effective ϵ_i:

$$\epsilon_i \approx -k_B T \ln(\langle n_i \rangle / \langle n_i^0 \rangle) \tag{52}$$

This enables the calculation of the entropy of counterion condensation, via Supplemental Equation 48, via the conversion of simulation data for n_i to the effective binding energy. This is the procedure used to calculate the values used in Figure 5 of the main manuscript.

Supplementary References

(1) Amblard, M.; Fehrentz, J.-A.; Martinez, J.; Subra, G. Methods and Protocols of Modern Solid Phase Peptide Synthesis. *Mol. Biotechnol.* **2006**, *33*, 239–254.

(2) Perry, S. L.; Leon, L.; Hoffmann, K. Q.; Kade, M. J.; Priftis, D.; Black, K. A.; Wong, D.; Klein, R. A.; Pierce, C. F. I.; Margossian, K. O.; Whitmer, J. K.; Qin, J.; de Pablo, J. J.; Tirrell, M. Chirality-Selected Phase Behaviour in Ionic Polypeptide Complexes. *Nat. Commun.* **2015**, *6*, 6052.

(3) Hoffmann, K. Q.; Perry, S. L.; Leon, L.; Priftis, D.; Tirrell, M.; de Pablo, J. J. A Molecular View of the Role of Chirality in Charge-Driven Polypeptide Complexation. *Soft Matter* **2015**, *11*, 1525–1538.

(4) Pacalin, N. M.; Leon, L.; Tirrell, M. Directing the Phase Behavior of Polyelectrolyte Complexes Using Chiral Patterned Peptides. *Eur. Phys. J. Spec. Top.* **2016**, *225*, 1805–1815.

(5) Priftis, D.; Laugel, N.; Tirrell, M. Thermodynamic Characterization of Polypeptide Complex Coacervation. *Langmuir* **2012**, *28*, 15947–15957.

(6) Lytle, T.K.; Radhakrishna, M.; Sing, C.E. High Charge Density Coacervate Assembly via Hybrid Monte Carlo Single Chain in Mean Field Theory. *Macromolecules* **2016**, *49,* 9693-9705.

(7) Hansen, J.P.; McDonald, I.R. *Theory of Simple Liquids.* Elsevier: Boston, MA **2006.**

(8) Frenkel, D.; Smit, B. *Understanding Molecular Simulation, 2nd ed.* Academic Press: San Diego, CA **2001.**

(9) Liu, S.; Muthukumar, M. Langevin Dynamics Simulation of Counterion Distribution Around Isolated Flexible Polyelectrolyte Chains. *J. Chem. Phys.* **2002,** *116,* 9975.
